# circ_0023461 Silencing Protects Cardiomyocytes from Hypoxia-Induced Dysfunction through Targeting miR-370-3p/PDE4D Signaling

**DOI:** 10.1155/2021/8379962

**Published:** 2021-10-01

**Authors:** Kai Ren, Buying Li, Liqing Jiang, Zhiheng Liu, Fan Wu, Yi Zhang, Jincheng Liu, Weixun Duan

**Affiliations:** ^1^Department of Cardiovascular Surgery, Xijing Hospital, Air Force Military Medical University, Xi'an, China; ^2^Department of Cardiovascular Surgery, Second Affiliated Hospital of Xi'an Jiaotong University, Xi'an, Shaanxi, China

## Abstract

**Background:**

Acute myocardial infarction (AMI) is a common cardiovascular disease with high disability and mortality. Circular RNAs (circRNAs) are implicated in the pathomechanism of multiple human diseases, including AMI. This study intended to explore the function and working mechanism of a novel circRNA circ_0023461 in hypoxia-induced cardiomyocytes.

**Methods:**

Reverse transcription-quantitative polymerase chain reaction (RT-qPCR) and Western blot assay were implemented to detect RNA and protein expression. Cell counting kit-8 (CCK8) assay and 5-ethynyl-2′-deoxyuridine (Edu) assay were conducted to analyze cell viability and proliferation ability. Cell migration and apoptosis were assessed by Transwell assay and flow cytometry. Cell oxidative stress was analyzed using the commercial kits. Enzyme-linked immunosorbent assay (ELISA) was conducted to analyze cell inflammation. Cell glycolytic metabolism was evaluated using the commercial kits. Dual-luciferase reporter assay and RNA pull-down assay were conducted to verify the intermolecular interactions.

**Results:**

circ_0023461 expression was upregulated in AMI patients and hypoxia-induced AC16 cells. Hypoxia restrained the viability, proliferation, migration, and glycolysis and induced the apoptosis, oxidative stress, and inflammation of AC16 cells, and these effects were attenuated by the silence of circ_0023461. MicroRNA-370-3p (miR-370-3p) was verified as a target of circ_0023461, and circ_0023461 silencing-mediated protective effects in hypoxia-induced cardiomyocytes were partly alleviated by the knockdown of miR-370-3p. miR-370-3p interacted with the 3′ untranslated region (3′ UTR) of phosphodiesterase 4D (PDE4D), and PDE4D overexpression partly reversed miR-370-3p overexpression-induced protective effects in hypoxia-induced cardiomyocytes. circ_0023461 can upregulate PDE4D expression by acting as a molecular sponge for miR-370-3p in AC16 cells.

**Conclusion:**

circ_0023461 knockdown attenuated hypoxia-induced dysfunction in AC16 cells partly by targeting the miR-370-3p/PDE4D axis.

## 1. Introduction

Acute myocardial infarction (AMI) is a common coronary artery disease with high disability and mortality globally, which is associated with cardiomyocyte dysfunction induced by acute ischemia-hypoxia and eventually leads to irreversible myocardial damage [1, 2]. Uncovering the molecular mechanism behind hypoxia-induced dysfunction in cardiomyocytes is important to develop novel effective clinical strategy for AMI patients.

Circular RNAs (circRNAs) are a novel class of noncoding RNAs characterized by closed circular structure without 5′ or 3′ end [3]. Therefore, circRNAs are resistant to exonuclease and exhibit high stability. circRNAs have tissue- or cell-specific expression pattern [4], and they are implicated in the regulation of multiple cellular biological behaviors [5]. All these characteristics make circRNAs ideal biomarkers for human diseases. circRNAs play crucial roles in human heart diseases [6, 7]. For instance, circ_0060745 silencing is reported to attenuate AMI progression by inactivating NF-*κ*B signaling [8]. A previous study uncovered that circ_0023461 level is elevated in the plasma samples of AMI patients compared with normal volunteers. In this study, we explored the role and working mechanism of circ_0023461 in hypoxia-induced cardiomyocytes.

MicroRNAs (miRNAs) are identified as pivotal regulators in multiple biological processes [9]. Accumulating evidence has pointed out that miRNAs regulate the progression of human cardiovascular diseases [10, 11]. Chu et al. found that miR-130 contributes to AMI-induced damage in cardiomyocytes by regulating PPAR-*γ* [12]. circRNAs can function as miRNA sponges to regulate cellular biological behaviors [13]. Through bioinformatics prediction, miR-370-3p was a possible target of circ_0023461. Zhao et al. found that miR-370 attenuates myocardial ischemia/reperfusion-induced injury in mice by regulating PLIN5-dependent PPAR signaling [14]. Here, we tested the interaction between miR-370-3p and circ_0023461, and their functional correlation in regulating hypoxia-induced injury in cardiomyocytes was further explored.

miRNAs can regulate gene expression by interacting with the 3′ untranslated region (3′ UTR) of target messenger RNAs (mRNAs) [15]. Phosphodiesterase 4D (PDE4D) was a predicted target of miR-370-3p by the bioinformatics database. A previous article reported that PDE4D polymorphisms are related to the pathogenesis of ischemic stroke [16]. Zhou et al. found that PDE4D expression is increased in myocardial infarction cell model, and PDE4D facilitates the apoptosis of myocardial cells [17]. In this study, we assessed the functional linkage between miR-370-3p and PDE4D in hypoxia-induced injury in cardiomyocytes.

We initially analyzed the role of circ_0023461 in hypoxia-induced cardiomyocytes. Subsequently, its downstream miRNA/mRNA signaling was predicted using the bioinformatics database and validated by rescue experiments.

## 2. Materials and Methods

### 2.1. Clinical Blood Samples

The blood samples were obtained from thirty AMI patients and thirty normal volunteers at Xijing Hospital, Air Force Military Medical University. The plasma supernatant samples were obtained through centrifuging at 1000 × g for 40 min at 4°C. Patients with severe liver or renal function defects, infections, malignancies, cardiomyopathies, and hematological diseases were excluded in this assay. All participants had signed written informed consent. All procedures in clinical study were approved by the Ethics Committee of Xijing Hospital, Air Force Military Medical University.

### 2.2. Cell Culture

Human cardiomyocyte AC16 cell line purchased from BeNa Culture Collection (Beijing, China) was cultured in Dulbecco's modified Eagle's medium (DMEM; Gibco, Carlsbad, CA, USA) plus 10% fetal bovine serum (FBS, Gibco) and 1% penicillin-streptomycin (Sigma, St. Louis, MO, USA) at 37°C with 5% CO_2_.

### 2.3. Model Establishment

AC16 cells were cultured under hypoxic condition in an incubator filled with 1% O_2_, 5% CO_2_, and 94% N_2_ for 24 h followed by normoxia condition filled with 21% O_2_, 5% CO_2_, and 74% N_2_ for 6 h to simulate clinical myocardial ischemia. AC16 cells under normoxia condition all the time were regarded as the control group.

### 2.4. Real-Time Quantitative Polymerase Chain Reaction (RT-qPCR)

RNA samples were obtained via commercial TRIzol reagent (Invitrogen, Carlsbad, CA, USA). Reverse transcription of miRNA was implemented using miRNA-specific stem-loop primer (RiboBio, Guangzhou, China), and complementary DNA (cDNA) of circRNA and mRNA was obtained using TaqMan Reverse Transcription Reagent (Invitrogen). qPCR was performed using SYBR Green (Takara, Dalian, China). U6 and glyceraldehyde 3-phosphate dehydrogenase (GAPDH) were the reference genes. The relative fold change was evaluated by the method of 2^-∆∆Ct^. All primers were presented in Table 1.

### 2.5. Verification of the Circular Characteristic of circ_0023461

RNAs (3 *μ*g) were digested with 9 U RNase R for 50 min. RT-qPCR was conducted to evaluate the resistance of circ_0023461 to RNase R.

### 2.6. Cell Transfection

Small interfering RNA of circ_0023461 (si-circ_0023461), negative control siRNA (si-NC), miR-370-3p mimics (miR-370-3p), control (miR-NC), inhibitor of miR-370-3p (anti-miR-370-3p), control (anti-miR-NC), PDE4D overexpression plasmid (PDE4D), and matched empty pcDNA vector (vector) were synthesized or constructed by GenePharma (Shanghai, China) and RiboBio. Lipofectamine 3000 reagent (Invitrogen) was adopted to transfect small RNAs or plasmids into AC16 cells.

### 2.7. Cell Counting Kit-8 (CCK8) Assay

AC16 cells were incubated with 20 *μ*L CCK8 reagent (Beyotime, Shanghai, China) for 4 h, and the optical density (OD) values at 450 nm were determined using the scan reader (BioTek, USA) to estimate cell viability.

### 2.8. 5-Ethynyl-2′-deoxyuridine (Edu) Assay

In brief, 20 *μ*M Edu solution (keyGEN Biotech, Jiangsu, China) was pipetted to the wells to mix with AC16 cells for 2 h, and cell nucleus was dyed using 4, 6-diamino-2-phenylindole dye liquor (DAPI; Sigma). The fluorescence pictures were taken using the fluorescence microscope.

### 2.9. Transwell Assays

A total of 200 *μ*L cell suspension (without serum) was pipetted to the upper compartments, and culture medium plus 10% FBS (chemokine) was pipetted to the lower compartments. After incubation for 24 h, unmigrated AC16 cells were scraped using the cotton swab, and cells crossed the membrane were dyed using 0.1% crystal violet dye liquor (Sangon Biotech, Shanghai, China). Five fields at 100x were randomly selected. The number of migrated cells was manually counted under an optical microscope (Olympus, Osaka, Japan).

### 2.10. Flow Cytometry

AC16 cells were first dispersed in binding reagent (BD Biosciences, Heidelberg, Germany), and then, 5 *μ*L Annexin V-fluorescein isothiocyanate (Annexin V-FITC; BD Biosciences) and 5 *μ*L propidium iodide (PI; BD Biosciences) were added to incubate with AC16 cells. Finally, the apoptotic cells marked with positive FITC and positive/negative PI were captured by a FACS CantoII flow cytometer (BD Biosciences).

### 2.11. Analysis of Cell Oxidative Stress

Malondialdehyde (MDA) content and superoxide dismutase (SOD) activity were examined using their matched kits (Jiancheng Biotech, Nanjing, China).

### 2.12. Enzyme-Linked Immunosorbent Assay (ELISA)

The release of interleukin 6 (IL-6) and tumor necrosis factor *α* (TNF-*α*) was assessed using enzyme-linked immunosorbent assay (ELISA) kits (Beyotime).

### 2.13. Analysis of Cellular Glycolysis

The levels of ATP and lactate and the consumption of glucose were analyzed using ATP Colorimetric Assay kit (Biovision, Milpitas, California, USA), Lactate Assay Kit II (Biovision), and Glucose Uptake Colorimetric Assay kit (Biovision).

### 2.14. Western Blot Assay

Protein samples were acquired using radioimmunoprecipitation assay (RIPA) buffer (Beyotime) and then were loaded onto separating gel and transferred to a polyvinylidene fluoride (PVDF) membrane (Millipore, Billerica, MA, USA). After sealing with 5% (*w*/*v*) nonfat milk for 1 h, the membrane was incubated with primary antibodies (Abcam, Cambridge, MA, USA) containing anti-hexokinase 2 (anti-HK2, ab227198), anti-lactate dehydrogenase A (anti-LDHA, ab125683), anti-PDE4D (ab249652), and anti-*β*-actin (ab8227). The membrane was labeled with appropriate secondary antibody (Abcam), and protein bands were visualized using the enhanced chemiluminescent substrate (Amersham Biosciences, Piscataway, NJ, USA).

### 2.15. Bioinformatics Analysis

circ_0023461-miRNA interactions were predicted by starBase and CircInteractome databases, and miR-370-3p-mRNA interactions were sought by starBase database.

### 2.16. Dual-Luciferase Reporter Assay

The wild-type (WT) or mutant-type (MUT) fragment of circ_0023461 or PDE4D, including the putative or mutant binding sequence with miR-370-3p, was subcloned into pmirGLO luciferase vector (Promega, Madison, WI, USA) to generate circ_0023461 WT, circ_0023461 MUT, PDE4D 3′ untranslated region (3′ UTR) WT, and PDE4D 3′ UTR MUT. AC16 cells were introduced with luciferase reporter plasmids and miR-370-3p or miR-NC. The luciferase activities of Firefly and Renilla in different transfected groups were determined using the commercial dual-luciferase reporter assay system (Promega). The luciferase intensity of Firefly was assessed with the Renilla intensity as the reference.

### 2.17. RNA Pull-Down Assay

Biotinylated miR-370-3p was constructed to generate biotin-miR-370-3p, and biotinylated miR-NC (biotin-NC) was regarded as the control. Cell extracts (2 *μ*g) were incubated with 100 pmol biotinylated RNAs followed by the addition of agarose beads (Invitrogen) for 1 h. The enrichment of circ_0023461 and PDE4D in the precipitated complex was analyzed by RT-qPCR.

### 2.18. Statistical Analysis

GraphPad Prism 7.0 software (GraphPad, La Jolla, CA, USA) was employed for data analysis, and the analytical results were denoted as mean ± standard deviation (SD). The differences were assessed by Student's *t*-test (in two groups) or one-way analysis of variance (ANOVA) (in multiple groups). Differences were statistically significant when *P* < 0.05.

## 3. Results

### 3.1. circ_0023461 Expression Is Elevated in the Plasma Samples of AMI Patients and Hypoxia-Induced Cardiomyocytes

**Figure 1:**
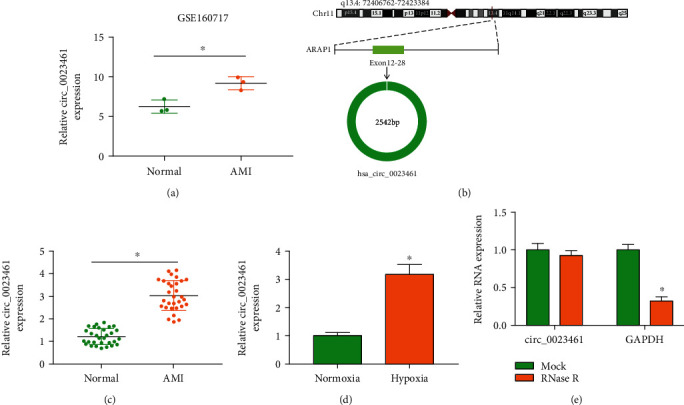
circ_0023461 expression is elevated in the plasma samples of AMI patients and hypoxia-induced cardiomyocytes. (a) The expression of circ_0023461 in the plasma samples of AMI patients (*n* = 3) and normal volunteers (*n* = 3) in the GSE160717 dataset was shown. (b) The genomic location and the structural characteristics of circ_0023461 were shown. circ_0023461 was derived from the exon 12-28 of ARAP1 gene. (c) RT-qPCR was conducted to measure the expression of circ_0023461 in the plasma samples of AMI patients (*n* = 30) and normal volunteers (*n* = 30). (d) The expression of circ_0023461 was examined in hypoxia-induced AC16 cells by RT-qPCR. (e) The resistance of circ_0023461 to RNase R was analyzed. ^∗^*P* < 0.05.

circ_0023461 expression was significantly elevated in the plasma samples of AMI patients (*n* = 3) compared with normal volunteers (*n* = 3) on the basis of the GSE160717 dataset (Figure 1(a)). We wondered whether dysregulated circ_0023461 was implicated in the regulation of AMI pathology. circ_0023461 was derived from the “back-splicing” of exon 12-28 of its host gene ARAP1 (Figure 1(b)). We collected the plasma samples of AMI patients (*n* = 30) and normal volunteers (*n* = 30) to further verify the expression pattern of circ_0023461. circ_0023461 expression was notably upregulated in the plasma samples of AMI patients compared with normal volunteers (Figure 1(c)). To explore the role of circ_0023461 in AMI *in vitro*, we established a myocardial ischemia cell model by exposing AC16 cells to hypoxia for 24 h followed by normoxia for 6 h. Hypoxia treatment significantly upregulated the expression of circ_0023461 in AC16 cells (Figure 1(d)). circ_0023461 was resistant to RNase R (Figure 1(e)), suggesting that circ_0023461 was indeed a circular transcript. These results suggested that circ_0023461 might be implicated in AMI progression.

### 3.2. circ_0023461 Silencing Protects Cardiomyocytes against Hypoxia-Induced Dysfunction

**Figure 2:**
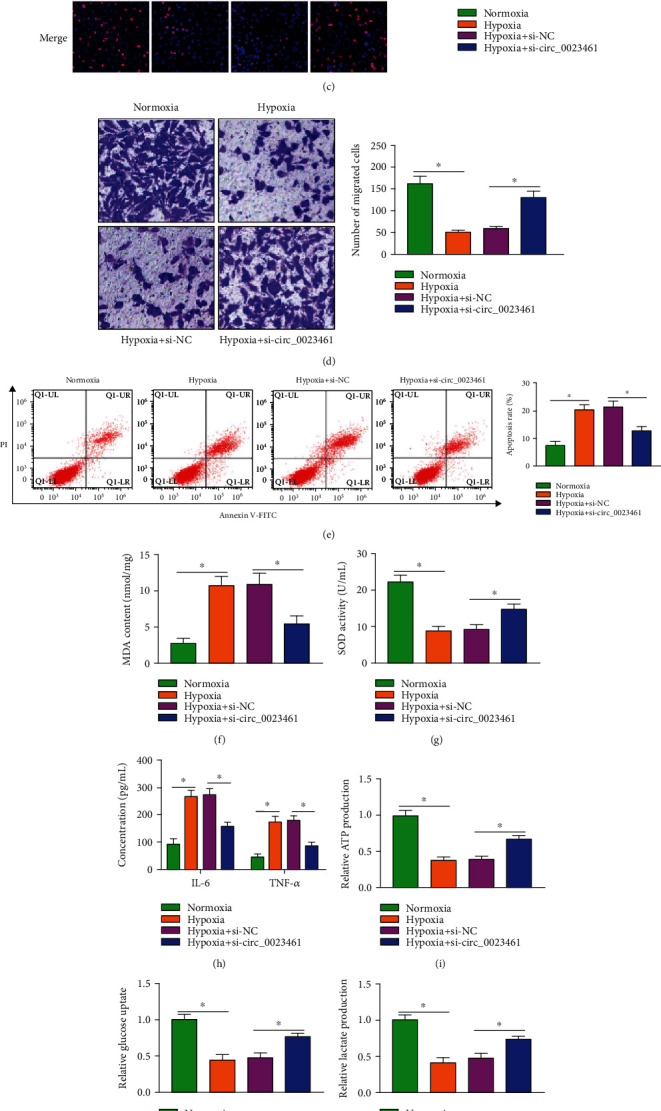
circ_0023461 silencing protects cardiomyocytes against hypoxia-induced dysfunction. (a) The transfection efficiency of si-circ_0023461 was analyzed in AC16 cells by RT-qPCR. (b–l) AC16 cells were divided into four groups: normoxia, hypoxia, hypoxia+si-NC, and hypoxia+si-circ_0023461. (b) Cell viability was analyzed by CCK8 assay. (c) Cell proliferation ability was evaluated by Edu assay. (d) Cell migration ability was assessed by Transwell migration assay. (e) Flow cytometry was performed to analyze cell apoptosis rate. (f, g) Cell oxidative stress was analyzed through measuring the content of MDA and the activity of SOD using the commercial kits. (h) The release of proinflammatory cytokines (IL-6 and TNF-*α*) was analyzed by ELISA assay. (i–k) Cell glycolytic metabolism was analyzed through detecting the uptake of glucose and the production of lactate and ATP using the colorimetric assay kits. (l) The protein levels of glycolysis-associated proteins (HK2 and LDHA) were measured by Western blot assay. ^∗^*P* < 0.05.

To explore the biological significance behind the abnormal upregulation of circ_0023461 in hypoxia-treated AC16 cells, we performed loss-of-function experiments. High silencing efficiency of si-circ_0023461 was verified by RT-qPCR assay (Figure 2(a)). Hypoxia treatment reduced cell viability (Figure 2(b)) and suppressed the proliferation (Figure 2(c)) and migration (Figure 2(d)) and induced the apoptosis (Figure 2(e)) of AC16 cells. Moreover, circ_0023461 knockdown partly attenuated hypoxia-induced dysfunction in AC16 cells (Figures 2(b)–2(e)). Then, we analyzed the role of circ_0023461 on the oxidative stress, inflammation, and glycolysis of hypoxia-induced AC16 cells. Hypoxia exposure induced the oxidative stress of AC16 cells, reflected by the elevated MDA level and reduced SOD activity (Figures 2(f) and 2(g)). The silence of circ_0023461 alleviated hypoxia-induced oxidative stress in AC16 cells (Figures 2(f) and 2(g)). Hypoxia treatment induced the release of proinflammatory cytokines, and cellular inflammatory response was partly attenuated by the silence of circ_0023461 (Figure 2(h)). Hypoxia exposure suppressed the production of ATP and lactate and the uptake of glucose, and these effects were all partly overturned by the knockdown of circ_0023461 (Figures 2(i)–2(k)). The levels of glycolysis-related key enzymes (HK2 and LDHA) were reduced in AC16 cells upon hypoxia treatment, and the silence of circ_0023461 partly rescued the expression of HK2 and LDHA (Figure 2(l)). These results suggested that circ_0023461 knockdown exerted a protective role in hypoxia-treated AC16 cells.

### 3.3. circ_0023461 Directly Interacts with miR-370-3p in Cardiomyocytes

**Figure 3:**
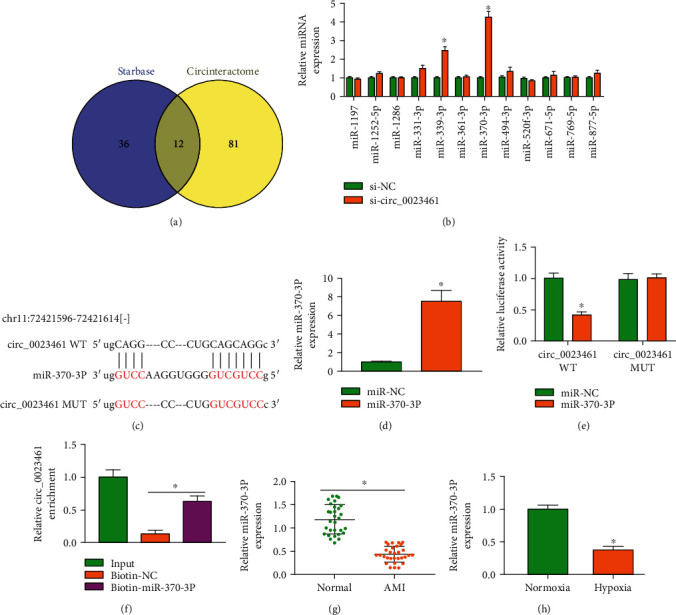
circ_0023461 directly interacts with miR-370-3p in cardiomyocytes. (a) The downstream miRNA targets of circ_0023461 were predicted by two bioinformatics databases, including starBase and CircInteractome. (b) The expression of 12 miRNAs in circ_0023461-silenced AC16 cells was measured by RT-qPCR. (c) The putative binding sequence between circ_0023461 and miR-370-3p was shown. (d) The transfection efficiency of miR-370-3p mimics in AC16 cells was analyzed by RT-qPCR. (e, f) Dual-luciferase reporter assay and RNA pull-down assay were conducted to validate whether miR-370-3p was a target of circ_0023461. (g) The level of miR-370-3p was examined in the plasma samples of AMI patients (*n* = 30) and normal volunteers (*n* = 30) by RT-qPCR. (h) RT-qPCR was performed to measure the expression of miR-370-3p in hypoxia-induced AC16 cells. ^∗^*P* < 0.05.

circRNAs can regulate cellular biological behaviors by acting as miRNA sponges [13]. We wondered whether circ_0023461 silencing-induced protective effects in hypoxia-induced AC16 cells were associated with miRNAs. Two online bioinformatics databases starBase (http://starbase.sysu.edu.cn) and CircInteractome (https://circinteractome.irp.nia.nih.gov) were used to predict the miRNA targets of circ_0023461. Venn diagram showed that there were 12 miRNAs that were predicted to be targets of circ_0023461 by both databases (Figure 3(a)). Among these 12 candidate miRNAs, the expression of miR-339-3p and miR-370-3p was significantly upregulated in circ_0023461-silenced AC16 cells (Figure 3(b)). The level of miR-370-3p in the circ_0023461-silenced group was more than 4 times higher than that in the si-NC group (Figure 3(b)). Therefore, we focused on the interaction between circ_0023461 and miR-370-3p. The putative binding sites between circ_0023461 and miR-370-3p were shown in Figure 3(c). High overexpression efficiency of miR-370-3p mimics was confirmed in AC16 cells (Figure 3(d)). Subsequently, dual-luciferase reporter assay and RNA pull-down assay were conducted to confirm the target relationship between circ_0023461 and miR-370-3p. After cotransfection with miR-370-3p instead of miR-NC, the luciferase activity of wild-type reporter plasmid (circ_0023461 WT) was significantly decreased (Figure 3(e)), suggesting the binding relationship between circ_0023461 and miR-370-3p. Meanwhile, the luciferase activity of mutant reporter plasmid (circ_0023461 MUT) remained unchanged when cotransfected with miR-NC or miR-370-3p (Figure 3(e)), indicating that the predicted sites were required for the interaction between circ_0023461 and miR-370-3p. RNA pull-down assay revealed that circ_0023461 was enriched when using biotinylated miR-370-3p (biotin-miR-370-3p) (Figure 3(f)), which further demonstrated the interaction between circ_0023461 and miR-370-3p. miR-370-3p expression was reduced in the plasma samples of AMI patients compared with normal volunteers (Figure 3(g)). Also, we found that hypoxia exposure significantly reduced the level of miR-370-3p in AC16 cells (Figure 3(h)). These results suggested that miR-370-3p was a direct target of circ_0023461 in AC16 cells.

### 3.4. circ_0023461 Silencing-Mediated Protective Effects in Hypoxia-Induced Cardiomyocytes Are Partly Overturned by the Knockdown of miR-370-3p

**Figure 4:**
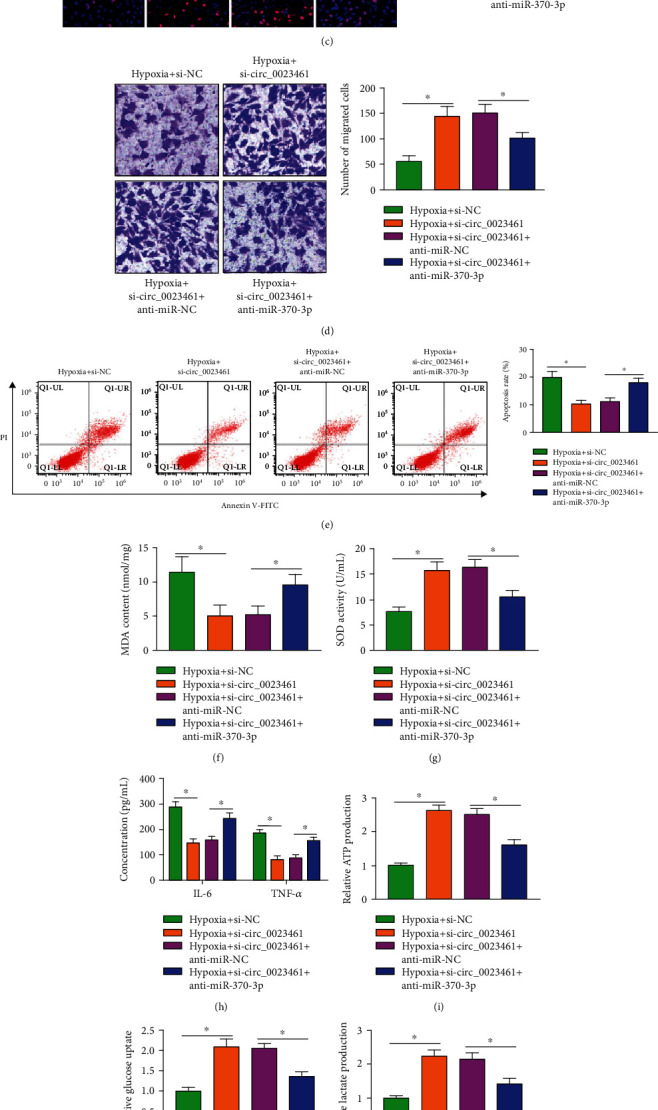
circ_0023461 silencing-mediated protective effects in hypoxia-induced cardiomyocytes are partly overturned by the knockdown of miR-370-3p. (a) RT-qPCR was conducted to analyze the transfection efficiency of anti-miR-370-3p in AC16 cells. (b–l) AC16 cells were divided into the following four groups: hypoxia+si-NC, hypoxia+si-circ_0023461, hypoxia+si-circ_0023461+anti-miR-NC, and hypoxia+si-circ_0023461+anti-miR-370-3p. (b) CCK8 assay was conducted to analyze cell viability. (c) Cell proliferation was analyzed by Edu assay. (d) Cell migration ability was analyzed by Transwell migration assay. (e) Cell apoptosis rate was evaluated by flow cytometry. (f, g) The content of MDA and the activity of SOD were measured using the commercial kits. (h) ELISA assay was conducted to analyze the production of proinflammatory cytokines (IL-6 and TNF-*α*) in the supernatant. (i–k) The consumption of glucose and the levels of lactate and ATP were measured using the colorimetric assay kits. (l) The protein levels of HK2 and LDHA were measured by Western blot assay. ^∗^*P* < 0.05.

We wondered whether the biological effects of circ_0023461 in hypoxia-induced cardiomyocytes were associated with miR-370-3p, and compensation experiments were conducted. RT-qPCR confirmed the high silencing efficiency of anti-miR-370-3p in AC16 cells (Figure 4(a)). AC16 cells were transfected with si-circ_0023461 alone or together with anti-miR-370-3p before hypoxia exposure. The introduction of anti-miR-370-3p suppressed cell viability, cell proliferation, and cell migration and induced cell apoptosis (Figures 4(b)–4(e)). Meanwhile, circ_0023461 silencing-induced effects on the oxidative stress, inflammation, and glycolytic metabolism were all partly reversed by the knockdown of miR-370-3p (Figures 4(f)–4(l)). These results together demonstrated that circ_0023461 silencing attenuated hypoxia-induced dysfunction in AC16 cells partly by upregulating miR-370-3p.

### 3.5. miR-370-3p Directly Binds to PDE4D in Cardiomyocytes

**Figure 5:**
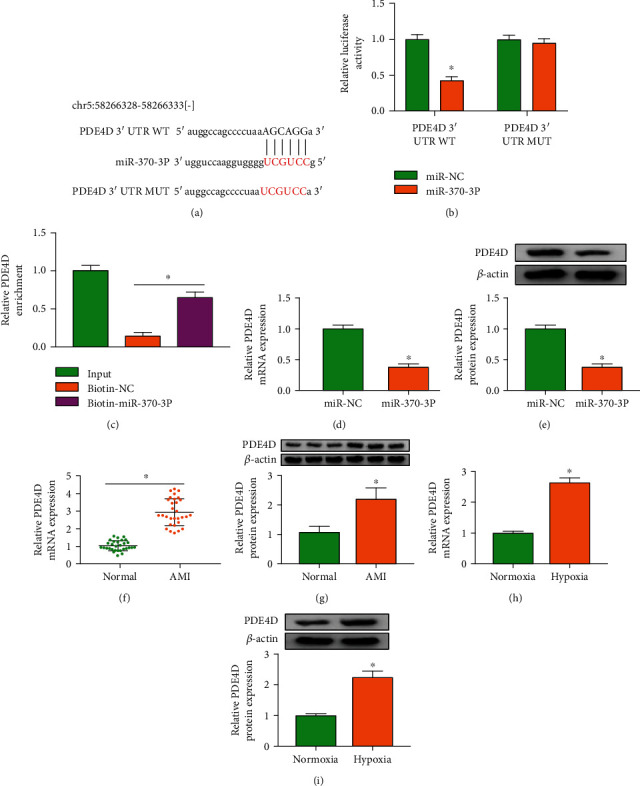
miR-370-3p directly binds to PDE4D in cardiomyocytes. (a) The interacted mRNAs of miR-370-3p were predicted by starBase database, and PDE4D was one of the candidate targets of miR-370-3p. (b, c) The binding relationship between miR-370-3p and PDE4D was verified by dual-luciferase reporter assay and RNA pull-down assay. (d, e) The effect of miR-370-3p overexpression on the mRNA and protein expression of PDE4D in AC16 cells was analyzed by RT-qPCR and Western blot assay. (f, g) The mRNA and protein expression of PDE4D was determined in the plasma samples of AMI patients and normal volunteers by RT-qPCR and Western blot assay. (h, i) The mRNA and protein levels of PDE4D were examined in hypoxia-treated AC16 cells by RT-qPCR and Western blot assay. ^∗^*P* < 0.05.

miRNAs can bind to the 3′ UTR of target mRNAs to induce their degradation or translational repression [15]. We predicted the possible mRNA targets of miR-370-3p using starBase database. PDE4D was one of the predicted targets of miR-370-3p, and their putative binding sites were shown in Figure 5(a). miR-370-3p overexpression markedly reduced the luciferase activity of wild-type reporter plasmid (PDE4D 3′ UTR WT) rather than mutant reporter plasmid (PDE4D 3′ UTR MUT) (Figure 5(b)), suggesting that miR-370-3p interacted with PDE4D via the predicted sequence. RNA pull-down assay further demonstrated the binding relationship between miR-370-3p and PDE4D (Figure 5(c)). miR-370-3p overexpression reduced the mRNA and protein expression of PDE4D in AC16 cells (Figures 5(d) and 5(e)). The mRNA and protein expression of PDE4D was increased in AMI patients and hypoxia-induced AC16 cells compared with normal volunteers and normoxia-treated AC16 cells (Figures 5(f)–5(i)). Overall, miR-370-3p negatively regulated PDE4D expression by directly binding to it in AC16 cells.

### 3.6. miR-370-3p Overexpression-Mediated Protective Effects in Hypoxia-Induced Cardiomyocytes Are Partly Counteracted by the Introduction of PDE4D Plasmid

**Figure 6:**
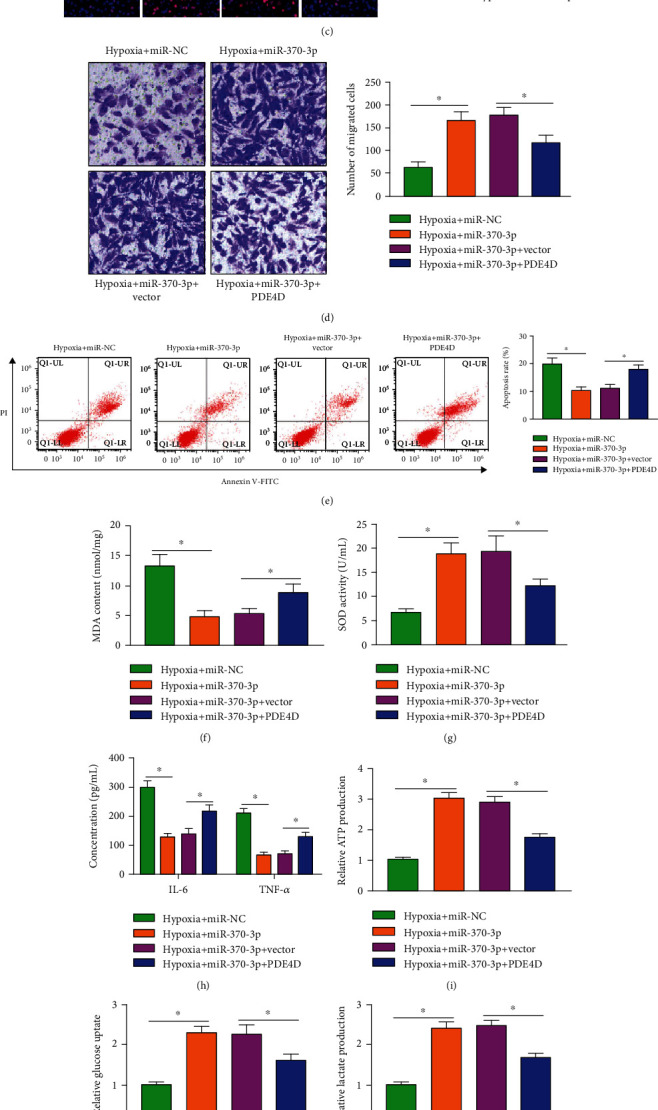
miR-370-3p overexpression-mediated protective effects in hypoxia-induced cardiomyocytes are partly counteracted by the introduction of PDE4D plasmid. (a) The transfection efficiency of PDE4D plasmid in AC16 cells was assessed by Western blot assay. (b–l) AC16 cells were divided into the following four groups: hypoxia+miR-NC, hypoxia+miR-370-3p, hypoxia+miR-370-3p+vector, and hypoxia+miR-370-3p+PDE4D. (b) CCK8 assay was conducted to assess cell viability. (c) Edu assay was performed to analyze the proliferation ability of AC16 cells. (d) Cell migration ability was assessed by Transwell migration assay. (e) The apoptosis of AC16 cells was analyzed by flow cytometry. (f, g) Cell oxidative status was analyzed using the matched kits. (h) The release of IL-6 and TNF-*α* was analyzed by ELISA. (i–k) The uptake of glucose and the production of lactate and ATP were analyzed using their matched colorimetric assay kits. (l) The expression of HK2 and LDHA was analyzed by Western blot assay. ^∗^*P* < 0.05.

To investigate whether miR-370-3p functioned by targeting PDE4D in hypoxia-induced cardiomyocytes, we performed compensation experiments through transfecting AC16 cells with miR-370-3p alone or together with PDE4D plasmid before hypoxia exposure. Western blot assay verified the high overexpression efficiency of PDE4D plasmid in AC16 cells (Figure 6(a)). miR-370-3p overexpression alone alleviated hypoxia-induced dysfunction in AC16 cells (Figures 6(b)–6(l)), which further demonstrated that miR-370-3p protected AC16 cells from hypoxia-induced injury. The addition of PDE4D plasmid reduced cell viability, suppressed cell proliferation and migration, and induced cell apoptosis (Figures 6(b)–6(e)). Moreover, PDE4D overexpression induced the oxidative stress and inflammation and suppressed the glycolytic metabolism of AC16 cells (Figures 6(f)–6(l)). These findings demonstrated that miR-370-3p overexpression protected AC16 cells from hypoxia-induced dysfunction partly by downregulating PDE4D.

### 3.7. PDE4D Is Regulated by circ_0023461/miR-370-3p axis in AC16 Cells

**Figure 7:**
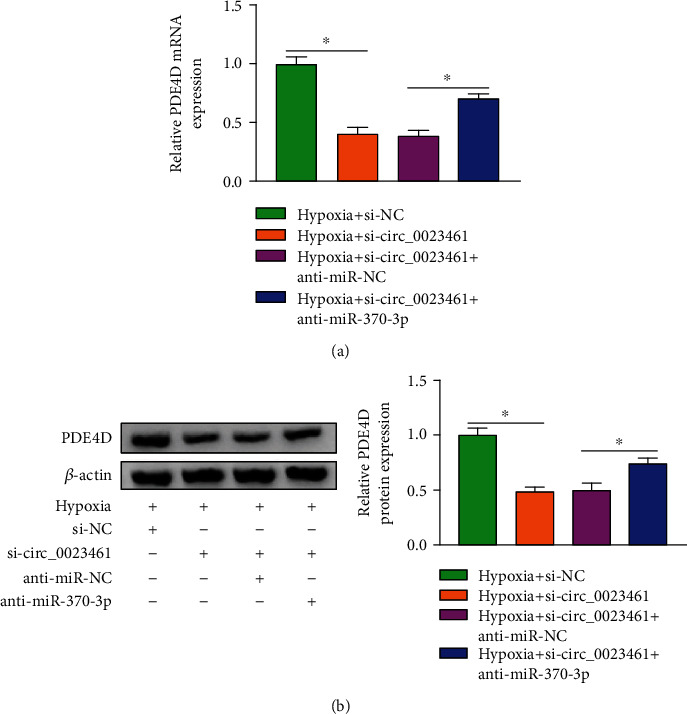
PDE4D is regulated by the circ_0023461/miR-370-3p axis in AC16 cells. (a, b) The mRNA and protein expression of PDE4D was examined in AC16 cells by RT-qPCR and Western blot assay in the following four groups: hypoxia+si-NC, hypoxia+si-circ_0023461, hypoxia+si-circ_0023461+anti-miR-NC, and hypoxia+si-circ_0023461+anti-miR-370-3p. ^∗^*P* < 0.05.

Considering the direct interaction between miR-370-3p and circ_0023461 or PDE4D, we further analyzed whether circ_0023461 regulated PDE4D expression by sponging miR-370-3p. circ_0023461 silencing markedly reduced PDE4D mRNA and protein expression, and its mRNA and protein levels were partly recovered by the addition of anti-miR-370-3p in AC16 cells (Figures 7(a) and 7(b)), suggesting that circ_0023461 positively regulated PDE4D expression by acting as a molecular sponge for miR-370-3p in AC16 cells.

## 4. Discussion

Accumulating evidence demonstrated the pivotal regulatory roles of circRNAs in ischemic heart diseases [6, 18, 19]. For instance, Liu et al. demonstrated that circ-ACAP2 level is elevated in the cardiomyocytes of a myocardial infarction rat model, and it promotes the apoptosis of cardiomyocytes after myocardial infarction by acting as a molecular sponge for miR-29 [20]. circ-Ttc3 is reported to exert a cardioprotective role by attenuating hypoxia-induced apoptosis and ATP depletion after myocardial infarction by absorbing miR-15b [21]. Therefore, circRNAs might serve as novel promising therapeutic targets for AMI. On the basis of the GSE160717 dataset, circ_0023461 level was significantly upregulated in AMI patients (*n* = 3) relative to normal volunteers (*n* = 3). Consistently, in our study, a distinct upregulation in the expression of circ_0023461 was found in AMI patients (*n* = 30) relative to healthy volunteers (*n* = 30).

We established a myocardial ischemia cell model by exposing cardiomyocytes AC16 to hypoxia for 24 h followed by normoxia for 6 h. Hypoxia exposure markedly elevated the expression of circ_0023461 in AC16 cells. We found that hypoxia exposure restrained the viability, proliferation, and migration and triggered the apoptosis, oxidative stress, and inflammatory response in cardiomyocytes. A previous article reported that the metabolic pathway of cardiomyocytes is changed during ischemia-related hypoxia due to the deficiency of oxygen and nutrient supply [22]. Therefore, we assessed the effect of hypoxia on the glycolytic metabolism of cardiomyocytes. We found that hypoxia stimulation markedly suppressed the glycolysis of cardiomyocytes. To explore the biological significance behind the aberrant upregulation of circ_0023461 in hypoxia-induced AC16 cells, we performed loss-of-function experiments. The results revealed that circ_0023461 interference alleviated hypoxia-mediated dysfunction in AC16 cells, demonstrating that hypoxia-induced dysfunction of cardiomyocytes was partly based on the up-regulation of circ_0023461.

Subsequently, we intended to analyze the working mechanism of circ_0023461 in the myocardial ischemia cell model. circRNAs have a gene regulatory potential by serving as miRNA sponges [13]. We predicted the downstream miRNA targets of circ_0023461 using two bioinformatics databases (starBase and CircInteractome). miR-370-3p was confirmed to be a downstream target of circ_0023461. miR-370-3p expression was decreased in AMI patients and hypoxia-induced AC16 cells. Previous studies demonstrated that miR-370-3p exerts a protective role in myocardial infarction. For instance, Zhang et al. demonstrated that circ_0010729 silencing attenuates hypoxia-induced dysfunction of cardiomyocytes by upregulating miR-370-3p [23], suggesting the protective role of miR-370-3p in myocardial infarction. Qiu et al. found that miR-370 suppresses hydrogen peroxide-induced oxidative stress and apoptosis in cardiomyocytes by regulating FOXO1 [24]. Zhao et al. found that miR-370 alleviates myocardial ischemia/reperfusion-induced injury in mice by regulating PLIN5-dependent PPAR signaling [14]. We found that miR-370-3p overexpression attenuated hypoxia-induced dysfunction in cardiomyocytes, which was consistent with former articles [14, 23, 24]. Moreover, circ_0023461 silencing-mediated protective effects in a myocardial ischemia cell model were partly counteracted by the silence of miR-370-3p, suggesting that circ_0023461 knockdown suppressed hypoxia-induced injury in cardiomyocytes partly by upregulating miR-370-3p.

miRNAs can bind to the 3′ UTR of target mRNAs to induce their degradation or translational repression [15]. PDE4D was identified as a downstream target of miR-370-3p in cardiomyocytes. PDE4D mRNA and protein expression was elevated in AMI patients and hypoxia-induced AC16 cells. Lehnart et al. found that PDE4D is implicated the regulation of cardiomyopathy progression and heart failure after myocardial infarction [25]. Zhou et al. claimed that PDE4D expression is upregulated in the myocardial infarction cell model, and PDE4D promotes the apoptosis of myocardial cells [17]. PDE4D overexpression largely counteracted miR-370-3p overexpression-mediated protective effects in the myocardial ischemia cell model, suggesting that miR-370-3p protected AC16 cells against hypoxia-induced dysfunction partly by downregulating PDE4D.

Considering the direct interaction between miR-370-3p and circ_0023461 or PDE4D, we explored whether circ_0023461 can indirectly regulate the expression of PDE4D by sponging miR-370-3p in AC16 cells. circ_0023461 silencing reduced the mRNA and protein expression of PDE4D, and the introduction of anti-miR-370-3p largely rescued the mRNA and protein expression of PDE4D, suggesting that circ_0023461 positively modulated PDE4D level by absorbing miR-370-3p in AC16 cells.

Some limitations existed in the current study. There was no data about the AMI rat or mouse model *in vivo*, and only one human cardiomyocyte cell line was used in this study. Therefore, the results may be unpersuasive to some extent. In future, human-induced pluripotent stem cell-derived atrial cardiomyocytes (hiPSC-aCMs) [26] need to be used to confirm the results in this study. In addition, rat cardiomyocyte cell line H9C2 and neonatal mouse cardiac myocytes need to be used to verify the role of the circ_0023461/miR-370-3p/PDE4D axis in other species *in vitro*, and AMI rat or mouse model *in vivo* needs to be established by ligating the coronary artery to further confirm the results in this study.

**Figure 8:**
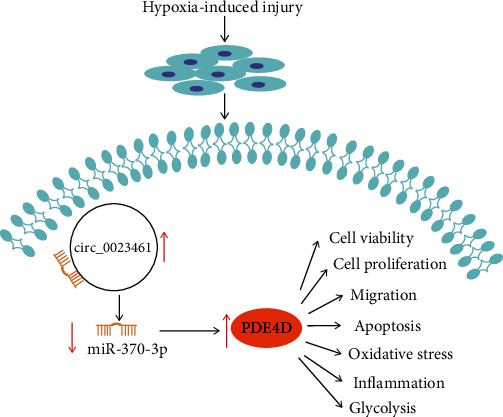
The role of the circ_0023461/miR-370-3p/PDE4D axis on hypoxia-induced AC16 cells.

In conclusion, the target relationship between miR-370-3p and circ_0023461 or PDE4D was confirmed in this study for the first time. circ_0023461 silencing alleviated hypoxia-induced dysfunction in cardiomyocytes by regulating the miR-370-3p/PDE4D axis (Figure 8), which provided a theoretical basis for the intervention of AMI.

## Figures and Tables

**Table 1:** Primers used in RT-qPCR.

Gene	Forward primer (5′-3′)	Reverse primer (5′-3′)
circ_0023461 miR-370-3p miR-1197 miR-1252-5p miR-1286 miR-331-3p miR-339-3p miR-361-3p miR-494-3p miR-520f-3p miR-671-5p miR-769-5p miR-877-5p PDE4D U6 GAPDH	ACGCTCTTCCAGACAGATGG GCCGAGGCCTGCTGGGGTGG GCCGAGTAGGACACATGGTC GCCGAGAGAAGGAAATTGAA GCCGAGTGCAGGACCAAGAT GCCGAGGCCCCTGGGCCTAT GCCGAGTGAGCGCCTCGACG GCCGAGTCCCCCAGGTGTGA GCCGAGTGAAACATACACGG GCCGAGAAGTGCTTCCTTTT GCCGAGAGGAAGCCCTGGAG GCCGAGTGAGACCTCTGGGT GCCGAGGTAGAGGAGATGGC CAGGGACTCAGGCGTTTTGA GCTTCGGCAGCACATATACTAAAAT CCTGTTCGACAGTCAGCCG	CTATTGTGCTGGGCAAGGA GCAGGGTCCGAGGTATTC GCAGGGTCCGAGGTATTC GCAGGGTCCGAGGTATTC GCAGGGTCCGAGGTATTC GCAGGGTCCGAGGTATTC GCAGGGTCCGAGGTATTC GCAGGGTCCGAGGTATTC GCAGGGTCCGAGGTATTC GCAGGGTCCGAGGTATTC GCAGGGTCCGAGGTATTC GCAGGGTCCGAGGTATTC GCAGGGTCCGAGGTATTC TGGCCAAGACCTGAGCAAAT CGCTTCACGAATTTGCGTGTCAT GAGAACAGTGAGCGCCTAGT

## Data Availability

Data are available on request.
